# Targeted Manipulation of Abundant and Rare Taxa in the *Daphnia magna* Microbiota with Antibiotics Impacts Host Fitness Differentially

**DOI:** 10.1128/mSystems.00916-20

**Published:** 2021-04-06

**Authors:** Reilly O. Cooper, Janna M. Vavra, Clayton E. Cressler

**Affiliations:** a School of Biological Sciences, University of Nebraska-Lincoln, Lincoln, Nebraska, USA; University of Connecticut; University of Wisconsin—Madison

**Keywords:** microbiome, invertebrate-microbe interactions

## Abstract

Host-associated microbes contribute to host fitness, but it is unclear whether these contributions are from rare keystone taxa, numerically abundant taxa, or interactions among community members. Experimental perturbation of the microbiota can highlight functionally important taxa; however, this approach is primarily applied in systems with complex communities where the perturbation affects hundreds of taxa, making it difficult to pinpoint contributions of key community members. Here, we use the ecological model organism Daphnia magna to examine the importance of rare and abundant taxa by perturbing its relatively simple microbiota with targeted antibiotics. We used sublethal antibiotic doses to target either rare or abundant members across two temperatures and then measured key host life history metrics and shifts in microbial community composition. We find that removal of abundant taxa had greater impacts on host fitness than did removal of rare taxa and that the abundances of nontarget taxa were impacted by antibiotic treatment, suggesting that no rare keystone taxa exist in the *Daphnia magna* microbiota but that microbe-microbe interactions may play a role in host fitness. We also find that microbial community composition was impacted by antibiotics differently across temperatures, indicating that ecological context shapes within-host microbial responses and effects on host fitness.

**IMPORTANCE** Understanding the contributions of rare and abundant taxa to host fitness is an outstanding question in host microbial ecology. In this study, we use the model zooplankton *Daphnia magna* and its relatively simple cohort of bacterial taxa to disentangle the roles of distinct taxa in host life history metrics, using a suite of antibiotics to selectively reduce the abundance of functionally important taxa. We also examine how environmental context shapes the importance of these bacterial taxa in host fitness.

## INTRODUCTION

The microbes in and on host organism tissue, collectively referred to as the microbiome, are recognized as having important beneficial impacts for the host. Many functions have been tied to bacterial species in the microbiota, including nutrient acquisition for the host (1) and immune system priming (2). As most species in host-associated microbiota are difficult to culture, experimental perturbation of the microbiota and subsequent sequencing combined with host fitness metric measurement are a commonly used set of methods to understand functional contributions of microbial taxa to host fitness (3–5). To understand the impacts of individual taxa on host fitness, antibiotics can be chosen to selectively perturb taxa and fitness outcomes can be measured (6). This approach is primarily used in systems with highly complex microbiomes, often with hundreds of interacting taxa impacted by these antibiotics (7, 8). While large-scale perturbations are necessary for understanding overall microbiome structure and broad-level interactions, fundamental questions about host-microbiome interactions can be addressed more readily in systems with simpler microbial communities. For example, determining whether host fitness is affected more by overall microbiome diversity (number of distinct taxa) or by functional diversity (taxa with distinct functions) is tractable in systems with fewer microbial taxa. Though interspecies interactions in the microbiome may complicate our understanding of how microbial taxa impact host fitness, identifying the contribution of taxa to host function is tractable in these simpler systems as subtle manipulation of the microbiome with sublethal doses of antibiotics can produce more easily understandable outcomes.

To better understand the relationship between specific taxa in the microbiota and host fitness, we applied an antibiotic suppression technique in Daphnia magna, a widely used model organism in ecotoxicology (9), population genomics (10), and host-parasite dynamics (11). The *Daphnia magna* microbiome is relatively simple, with only 10 to 15 amplicon sequence variants (ASVs) constituting >70% of relative abundance (12, 13). In particular, *Betaproteobacteria*, *Gammaproteobacteria*, and *Sphingobacteriia* are bacterial classes consistently identified in the *Daphnia magna* microbiome across environments and genotypes (14–16). Important contributions to host fitness may be directly linked to single taxa in these classes, as removal of the microbiota with broad-spectrum antibiotics has been directly linked to decreases in *Daphnia* growth, survival, and fecundity (13, 17, 18). In particular, *Limnohabitans*, a highly abundant genus of *Betaproteobacteria*, has been shown to benefit host fecundity (17). However, no host fitness benefits have been directly linked to the other bacterial classes prevalent in the *Daphnia magna* microbiome. In these cases, it is important to also consider that interspecies microbiome interactions impact host fitness (19). Taxa in the microbiome may have their functions mediated by other taxa, or cooccurrence may be beneficial for hosts and microbes (20), and as such, antibiotic suppression of one species in the relationship may indirectly impact the host by giving nonsusceptible microbial species competitive advantages or reducing fitness of codependent species that have functional importance to the host (21, 22).

Functions provided by the microbiota to the host can be dependent on biotic and abiotic factors. Environmental factors like temperature (23), pH (24), and food availability and diet (25) alter microbiome composition and gene expression profiles of present taxa. Intrinsic tolerance differences among taxa or host-mediated selection for tolerant taxa may drive changes in community composition, which in turn could influence host fitness. We aimed to investigate this environmental factor-microbiome-host fitness interaction using temperature, because *Daphnia magna* lives at a wide range of temperatures (26) and because temperature influences the *Daphnia magna* microbiota (12, 27). Here, we sought to understand which taxa were affected by environmental change using a cold, environmentally relevant temperature similar to that found in late fall and early spring (11°C) and a temperature in the center of the *Daphnia magna* range (19°C) (28), and whether impacted taxa contributed to host fitness.

To identify taxa in the *Daphnia* microbiota associated with specific host life history traits, we selected antibiotics with different modes of action that could perturb bacterial classes differentially. We used erythromycin (ERY), a macrolide that inhibits bacterial protein synthesis (29); aztreonam (AZT), a monobactam that inhibits bacterial cell wall synthesis (30); and sulfamethoxazole (SFX), a sulfonamide that interferes with bacterial folate biosynthesis (31). We particularly focused on the impacts of these antibiotics on the known abundant families in the *Daphnia magna* microbiota. Gram-negative, aerobic bacteria including *Betaproteobacteria*, *Gammaproteobacteria*, and *Sphingobacteriia* are susceptible to aztreonam (30). Some *Betaproteobacteria* are resistant to macrolides, including the presence of resistance genes in *Daphnia*-associated *Betaproteobacteria*, indicating that *Gammaproteobacteria* and *Sphingobacteriia* would be affected by erythromycin (32, 33). Sulfamethoxazole is primarily active against Gram-positive bacteria, but some Gram-negative taxa are susceptible, including some *Betaproteobacteria* and *Sphingobacteriia* (34, 35). We aimed to understand how antibiotics with different modes of action and different susceptible taxa changed the microbiome and impacted host fitness, linking changes in relative abundance to host fitness outcomes. Because the betaproteobacterium *Limnohabitans* impacts host fitness (17) and is present in high relative abundance in the *Daphnia magna* microbiota, we hypothesized that more abundant taxa contributed a greater share of functions impacting host fitness; as such, suppression of these more abundant taxa would reduce host fitness, specifically in reduced fecundity, survival, and growth. We also aimed to understand how the microbiome and its associated functions changed depending on environmental context by raising *Daphnia* in cold temperatures. We hypothesized the differing environment would induce shifts in microbiota composition, as different microbial functions may be necessary to respond to the change. Finally, we combined environmental change with antibiotic treatments to see if reduction of abundant taxa caused differential shifts in host fitness across environments. Here, we hypothesized that reduction of taxa abundant in warmer temperatures with antibiotics would not cause as severe changes in host fitness in the colder temperature treatment, as taxa providing beneficial functions in a different environment would not be targeted by these perturbations and would have reduced competition from the now-suppressed taxa.

## RESULTS

**FIG 1:**
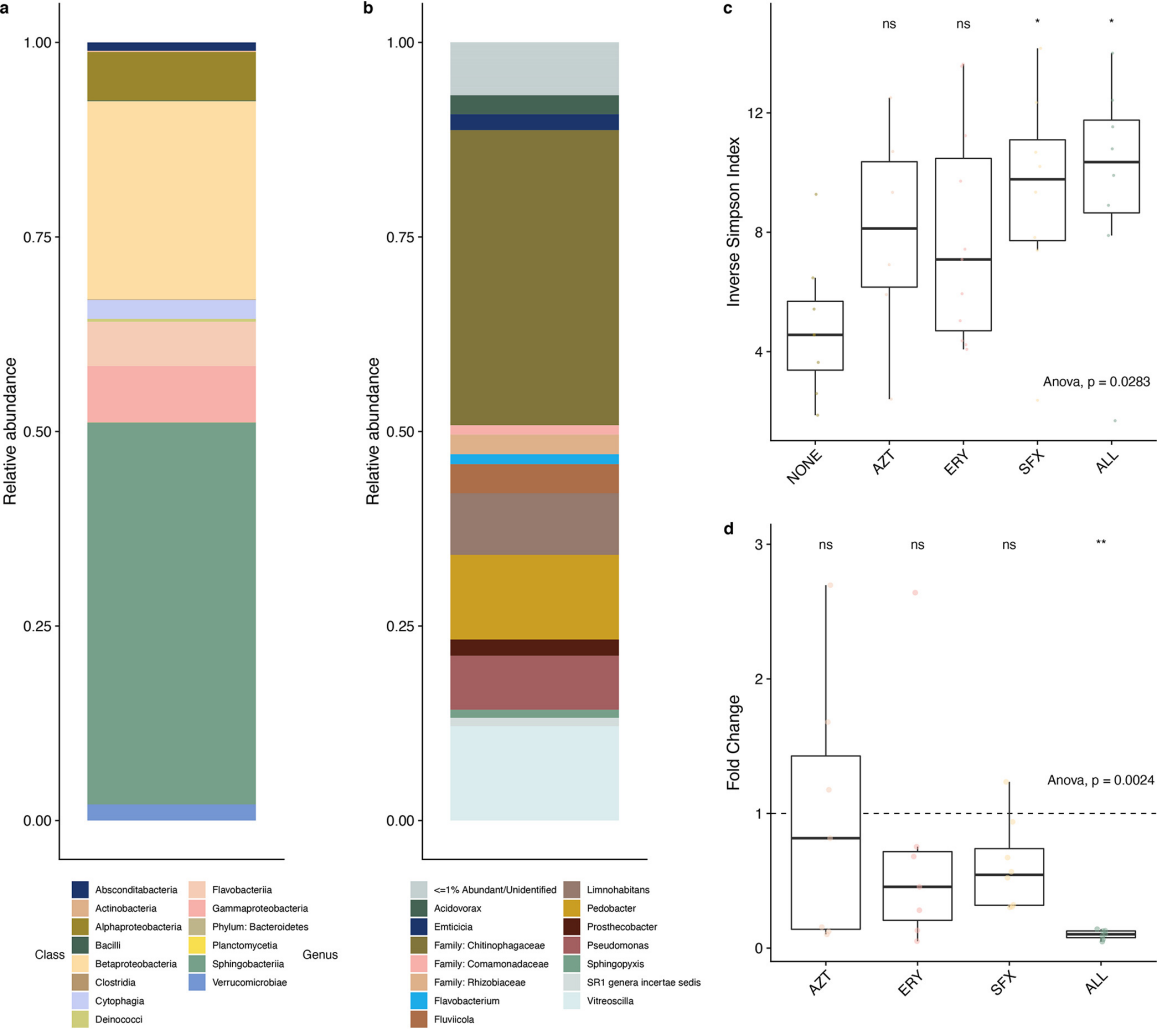
16S rRNA sequencing results from adult *Daphnia magna* under control conditions. (a) Identified classes in the *Daphnia magna* microbiota (if unidentified at the class rank, identified at the phylum rank). (b) Relative abundances of bacterial genera identified in the *Daphnia magna* microbiota (if unidentified at the genus rank, identified at the family rank). Taxa unidentifiable at or below family rank or less than 1% of total relative abundance are marked as <=1% Abundant/Unidentified. (c) Alpha diversity (inverse Simpson index) in all data across antibiotic treatments (ns, not significant; *, *P* < 0.05). (d) Box plot of bacterial relative abundance fold change in all data across antibiotic treatments compared to the control treatment, measured by amplification of the 16S rRNA V4 hypervariable region using qPCR (ns, not significant; **, *P* < 0.01). The horizontal line at fold change of 1 denotes the expected measurement of this amplified region in the no-antibiotic control.

The *Daphnia magna* microbiota was relatively simple, with only 8 bacterial classes of the 14 identified as more than 1% abundant (Fig. 1a). Of these, *Sphingobacteriia* and *Betaproteobacteria* were most common (48% and 26%, respectively). Only 10 unique ASVs comprised approximately 60% of total abundance (Fig. 1b). Primarily, the most abundant ASVs belonged to the *Limnohabitans*, *Pedobacter*, and *Vitreoscilla* genera, and one ASV unidentified below the *Chitinophagaceae* family rank was highly abundant.

**FIG 2:**
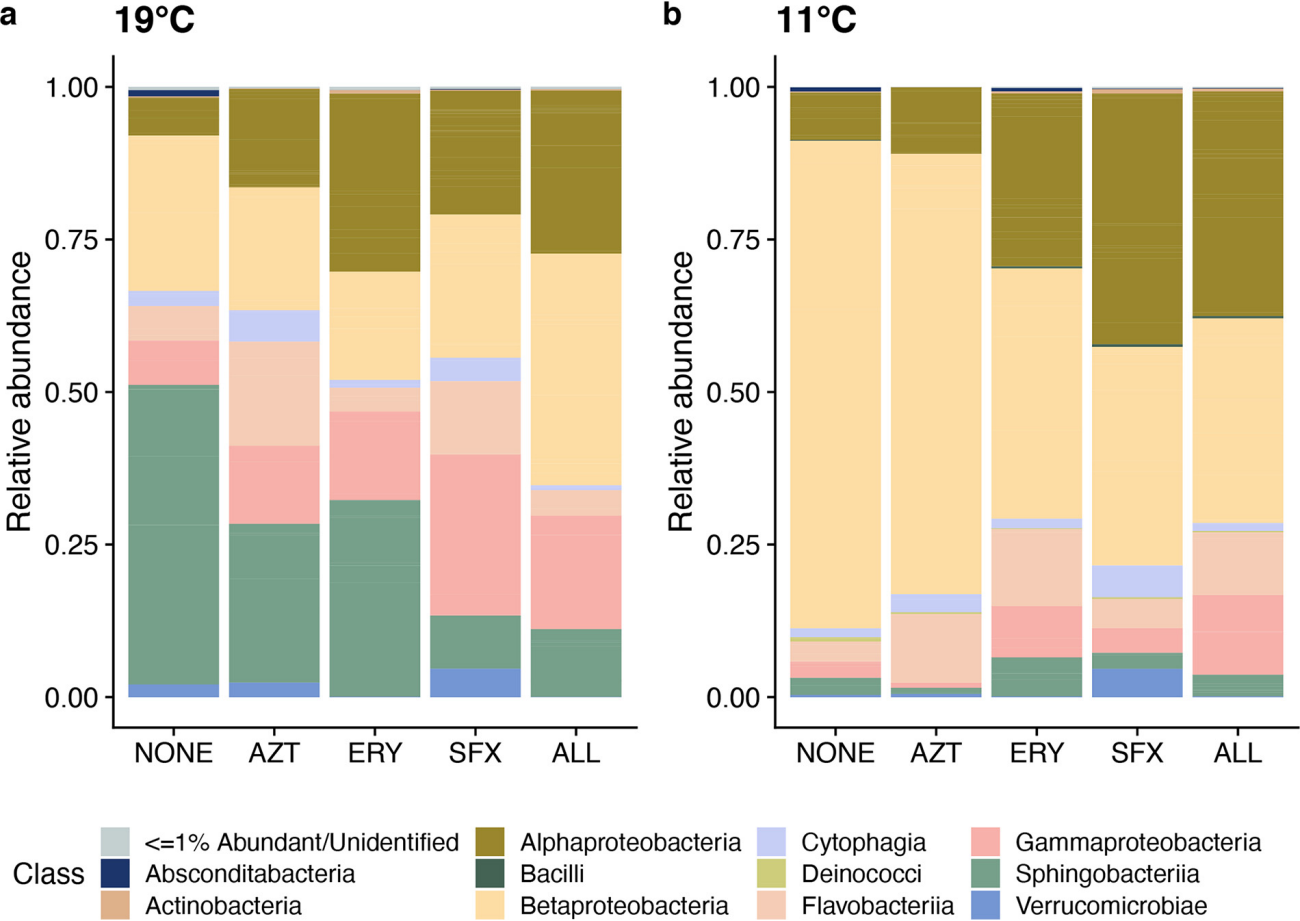
Microbiota composition at the class level in *Daphnia magna* across antibiotic treatments and across temperature treatments (NONE = no antibiotics; AZT = aztreonam; ERY = erythromycin; SFX = sulfamethoxazole; ALL = AZT, ERY, and SFX). Taxa are conglomerated at the class rank to show differences in relative abundance of taxa among antibiotic treatments.

**FIG 3:**
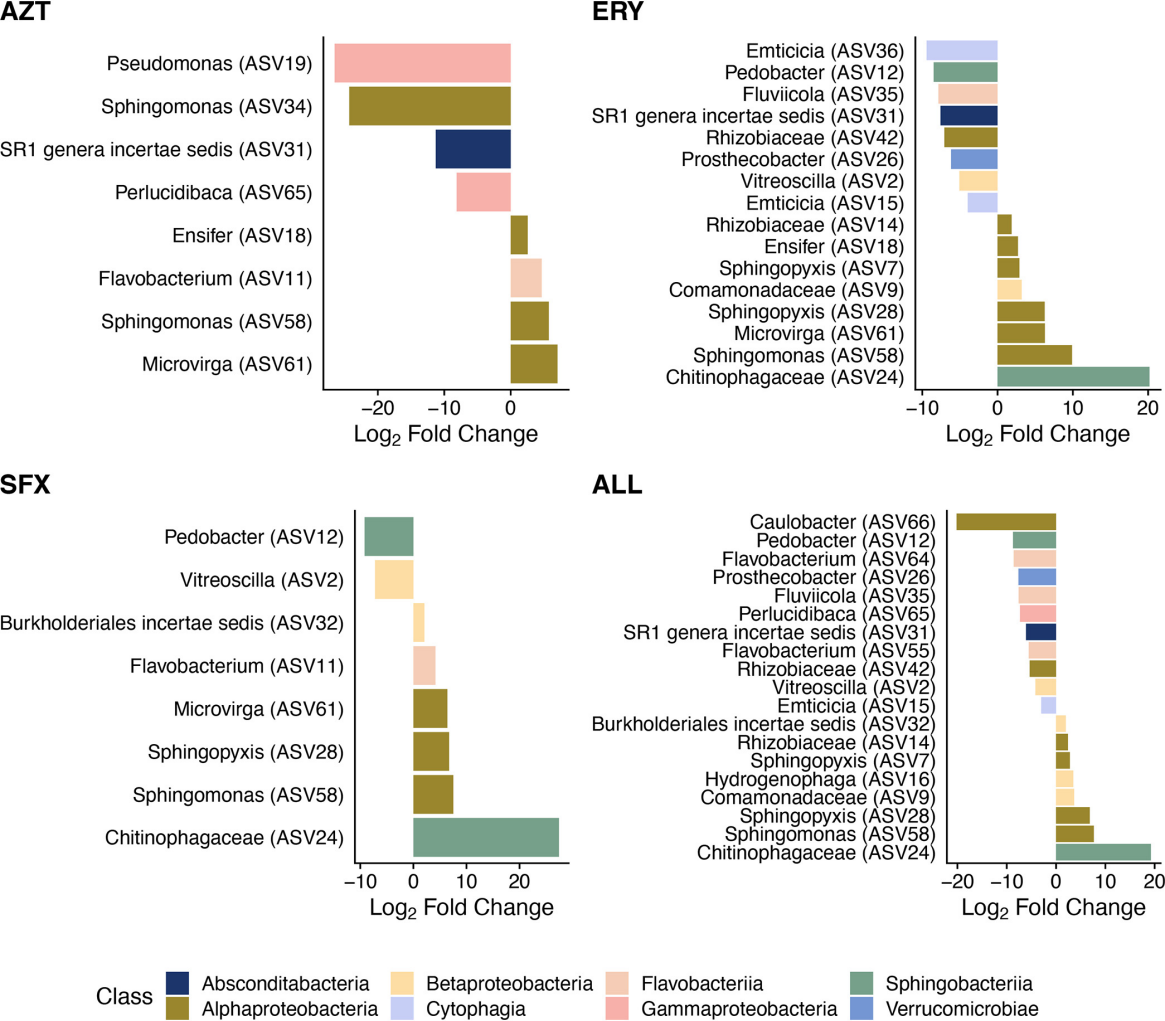
Differentially abundant ASVs (α < 0.05) in each antibiotic treatment compared to the no-antibiotic control in the 19°C treatment after 21 days of treatment. Each bar represents a single ASV identified to the genus level, with genus name indicated on the left. Bar color indicates the bacterial class of each ASV, and bar length indicates the fold change in abundance of each ASV.

Antibiotic treatment significantly impacted alpha diversity (*F*_4,31_ = 3.133, *P* = 0.028; Fig. 1c), with both sulfamethoxazole and the antibiotic trio having significantly higher alpha diversity than the no-antibiotic control (both *P* < 0.05, Tukey honestly significant difference [HSD]). Relative abundance of bacteria in *Daphnia magna* was significantly impacted by antibiotic treatment (*F*_4,32_ = 5.197, *P* = 0.0024; Fig. 1d). Mean change in bacterial load in *Daphnia* treated with the antibiotic trio was 0.1-fold relative to the baseline abundance of control *Daphnia*, while treatment with each of the three antibiotics individually slightly reduced relative abundance (AZT, 0.96-fold change; ERY, 0.71-fold change; SFX, 0.61-fold change). The microbiota was significantly impacted by antibiotic treatments at the class rank (permutational multivariate analysis of variance [PERMANOVA], pseudo-*F*_4,41_ = 3.977, *R*^2^ = 0.26, *P* = 0.01; Fig. 2). Pairwise PERMANOVA comparisons of antibiotic treatments to the no-antibiotic control indicated significant impacts of erythromycin (*P* = 0.01), sulfamethoxazole (*P* = 0.001), and the antibiotic trio (*P* = 0.001) on microbiota composition (see Table S1A in the supplemental material). We found multiple differentially abundant ASVs in each antibiotic treatment (Fig. 3, Table S1B). Though aztreonam-treated samples did not have significantly different overall composition than the control (Table S1A), there were differences in the relative abundance of 8 ASVs, including decreases in *Pseudomonas* (ASV 19, 2^−26.5^, or 10^−8^ lower *Pseudomonas* relative abundance than in the no-antibiotic control) and *Sphingomonas* (ASV 34, 2^−24.3^) and increases in *Microvirga* (ASV 61, 2^7.04^). Erythromycin had 16 differentially abundant ASVs, with an unidentified *Chitinophagaceae* (ASV 24) and *Emticicia* (ASV 36) experiencing the greatest fold abundance changes (2^20.2^ and 2^−9.43^, respectively). Treatment with sulfamethoxazole induced changes for 8 ASVs, primarily increasing the abundance of the *Chitinophagaceae* ASV (ASV 24, 2^27.4^) and decreasing the abundance of *Pedobacter* (ASV 12, 2^−9.19^). Treatment with the antibiotic trio impacted the abundances of 19 ASVs, contributing to fold increases of the same *Chitinophagaceae* (ASV 24, 2^19.24^) and decreasing the abundance of *Caulobacter* (ASV 66, 2^−20.18^). In summary, aztreonam reduced the relative abundances of *Gammaproteobacteria* and some *Alphaproteobacteria*; erythromycin increased the relative abundance of *Alphaproteobacteria* while decreasing the relative abundances of multiple other classes including *Gammaproteobacteria* and *Sphingobacteriia*; and sulfamethoxazole decreased the relative abundance of *Betaproteobacteria* and increased *Alphaproteobacteria*. The antibiotic trio had the most wide-ranging effects on the microbiota, increasing the relative abundance of some *Sphingobacteriia* but primarily decreasing relative abundances across multiple classes.

10.1128/mSystems.00916-20.3 TABLE S1 (A) Pairwise PERMANOVA comparisons of the *Daphnia magna* microbiome across antibiotic treatments compared to the no-antibiotic control. Bolded values indicate *P* < 0.05. (B) Differentially abundant taxa in the *Daphnia magna* microbiota in the 19°C treatment across antibiotic treatments compared to the no-antibiotic control (α < 0.05), as determined by DESeq2 analysis. Bolded values indicate *P* < 0.05. (C) *Post hoc* Tukey HSD of cumulative *Daphnia magna* reproduction across antibiotic treatments and temperature treatments. (D) *Post hoc* Tukey HSD of first brood size (number of offspring) for *Daphnia magna* across antibiotic treatments. (E) *Post hoc* Tukey HSD of *Daphnia magna* growth across antibiotic treatments and temperature treatments. Download Table S1, XLSX file, 0.03 MB. Copyright © 2021 Cooper et al. 2021 Cooper et al. https://creativecommons.org/licenses/by/4.0/ This content is distributed under the terms of the Creative Commons Attribution 4.0 International license.

The *Daphnia magna* microbiota was significantly impacted by temperature. In the no-antibiotic control, composition was significantly different across temperatures (pseudo-*F*_1,6_ = 5.142, *R*^2^ = 0.46, *P* = 0.03; Table S1A). In all antibiotic treatments except aztreonam, there were significant differences in microbiota composition between temperatures compared to the control (pairwise PERMANOVAs, erythromycin pseudo-*F*_1,9_ = 4.001, *R*^2^ = 0.31, *P* = 0.006; sulfamethoxazole pseudo-*F*_1,9_ = 2.605, *R*^2^ = 0.30, *P* = 0.045; trio pseudo-*F*_1,6_ = 5.142, *R*^2^ = 0.46, *P* = 0.034; Table S1A). Multiple taxa were found to be differentially abundant in each antibiotic treatment across both temperatures (Fig. S1, Table S1B). The no-antibiotic control had a single ASV (*Sphingobacteriia* genus, 2^−13.68^) that was differentially abundant in cold temperatures. In aztreonam, 6 ASVs were reduced in relative abundance in cold temperatures, with a *Sphingobacteriia* ASV (unidentifiable beyond class) and an *Alphaproteobacteria* genus experiencing the greatest reductions (2^−11.98^ and 2^−11.4^, respectively). In erythromycin, 12 ASVs were differentially abundant across temperatures; of those, the same *Sphingobacteriia* ASV as in the aztreonam treatment was reduced (2^−27.54^) in 11°C and a *Pseudomonas* ASV was more abundant (2^6.34^) in 11°C. Sulfamethoxazole impacted 13 ASVs, including *Limnohabitans* (2^−6.61^) and the same *Sphingobacteriia* ASV (2^−25.84^). The antibiotic trio treatment had only one differentially abundant ASV (*Nubsella*, 2^7.89^). Generally, differentially abundant classes were limited to the *Alphaproteobacteria*, *Betaproteobacteria*, *Flavobacteriia*, and *Sphingobacteriia*. *Sphingobacteriia* exhibited the greatest changes in abundance across antibiotic treatments, with a *Sphingobacteriia* genus experiencing between 2^−5^ and 2^−27.5^ reduced abundance in no antibiotics, aztreonam, erythromycin, and sulfamethoxazole at low temperatures as compared to those treatments in the control temperature.

10.1128/mSystems.00916-20.1 FIG S1 Differentially abundant ASVs (α < 0.05) in each antibiotic treatment at 11°C compared to the same antibiotic treatment in the 19°C treatment after 21 days of treatment. Each bar represents a single ASV identified to the genus level, with genus name indicated on the left. Bar color indicates the bacterial class of each ASV, and bar length indicates the fold change in abundance of each ASV. Download FIG S1, TIF file, 2.1 MB. Copyright © 2021 Cooper et al. 2021 Cooper et al. https://creativecommons.org/licenses/by/4.0/ This content is distributed under the terms of the Creative Commons Attribution 4.0 International license.

**FIG 4:**
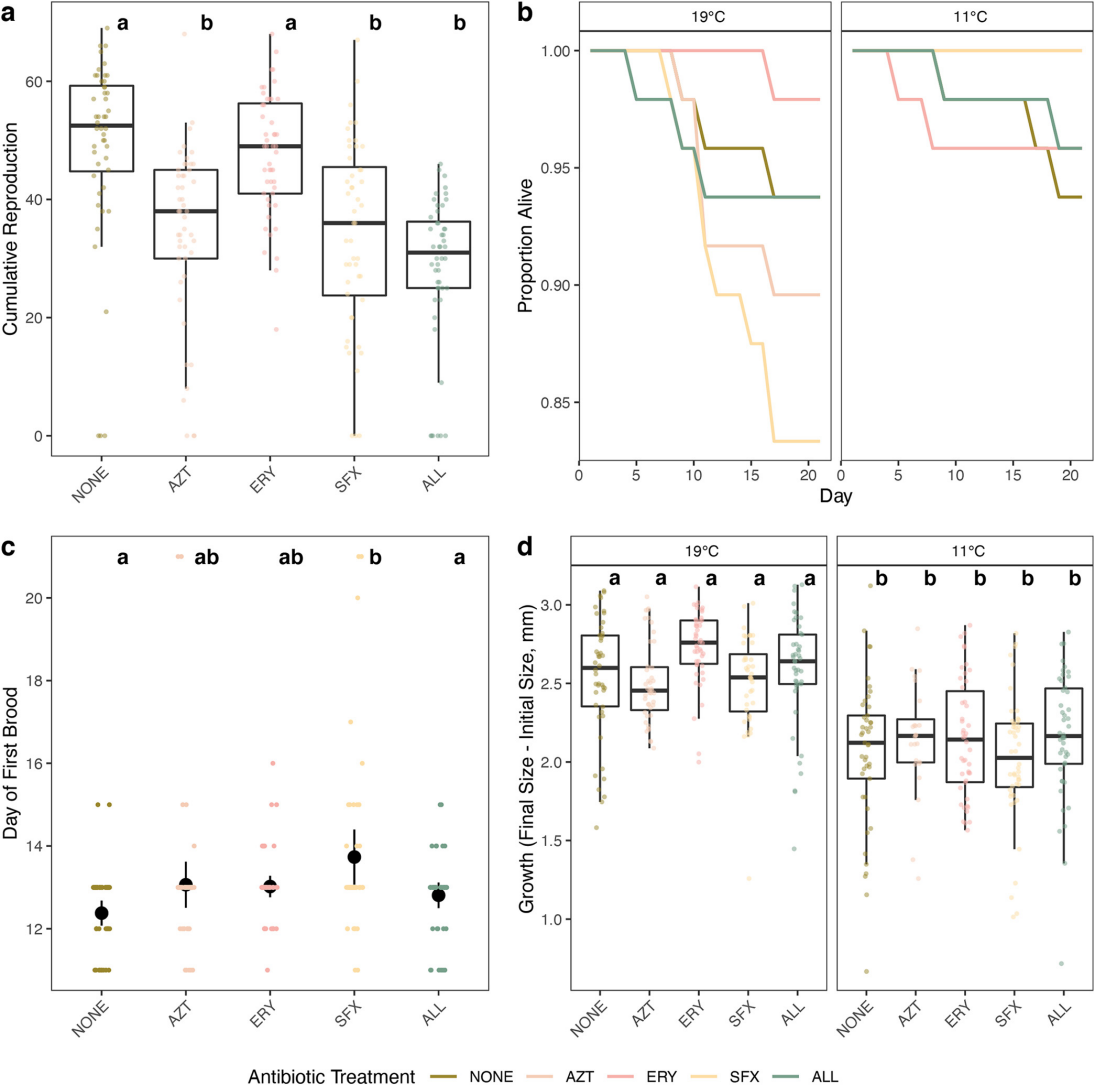
Summary of *Daphnia magna* fitness. (a) Box plot of cumulative reproduction over the 21-day experiment in *Daphnia magna* across antibiotic treatments (NONE = no antibiotics, AZT = aztreonam, ERY = erythromycin, SFX = sulfamethoxazole, ALL = all three antibiotics) in the 19°C temperature treatment. Points show cumulative reproduction of each individual *Daphnia* over the 21-day experiment. Letters denote significant differences among treatments. (b) Survival curves of *Daphnia magna* in antibiotic treatments across temperature treatments. Line color and pattern denote antibiotic treatment. (c) Time to reproductive maturity of *Daphnia magna* in the 19°C temperature treatment across antibiotic treatments. Jittered points denote individuals within each antibiotic treatment. Letters denote significant differences among treatments. (d) *Daphnia magna* growth in millimeters over the 21-day experiment across antibiotic and temperature treatments for individuals who survived the entire time course. Box plots denote median and first and third quartiles, and jittered points show growth of each individual within a treatment.

Host fitness was significantly impacted by antibiotics. In particular, cumulative host reproduction over the course of the experiment was reduced by antibiotics (*F*_4,470_ = 49.59, *P* < 0.001). *Post hoc* Tukey tests revealed that this reduction was most significant in the aztreonam, sulfamethoxazole, and antibiotic trio treatments (all *P* < 0.001) (Fig. 4a). A complete list of *post hoc* comparisons for cumulative reproduction can be found in Table S1C. Though cumulative host reproduction was reduced in these treatments and many amplicon sequence variants experienced shifts in relative abundance across treatments, there was no single bacterial genus that shifted in relative abundance predictably across treatments, suggesting that there is no genus that is uniquely important to *D. magna* reproductive fitness. Reproductive timing was also impacted (*F*_4,222_ = 4.797, *P* < 0.001), where *Daphnia magna* treated with sulfamethoxazole experienced a later age at first reproduction than those treated with other antibiotics (Tukey HSD, *P* = 0.03; Fig. 4c and Table S1D). *Daphnia magna* exposed to antibiotics experienced a significant overall reduction in growth (ANOVA, *F*_4,419_ = 2.08, *P* = 0.004; Table S1E); the main contributor to this was a significant reduction in growth in sulfamethoxazole compared to erythromycin (Tukey HSD, *P* = 0.003; Table S1E). Exposure to any antibiotic had no impact on *Daphnia* survival (Fig. 4b and Fig. S2). Host fitness was also impacted by temperature. Cumulative reproduction was reduced almost completely in the cooler 11°C treatment; no reproduction was observed in the control and only one adult individual reproduced across all of the antibiotic treatments (ANOVA, *F*_1,470_ = 1,751.08, *P* < 0.001), hence Fig. 4a and c visualizing data from only the 19°C treatment. There was also an effect of the interaction between antibiotics and temperature (ANOVA, *F*_4,470_ = 19.84, *P* < 0.001), though no effects of antibiotics on reproduction were observed due to the strong effect of temperature. *Daphnia* growth was limited in the cold-temperature treatment (*F*_1,419_ = 176.01, *P *< 0.001) (Fig. 4d). Temperature impacted survival, with *Daphnia* in colder temperatures surviving significantly more than those in control temperatures (hazard ratio = −1.087, *P* = 0.013) (Fig. 4b and Fig. S2).

10.1128/mSystems.00916-20.2 FIG S2 Survival hazard ratios for *Daphnia magna* in each antibiotic treatment and temperature treatment. Download FIG S2, TIF file, 1.2 MB. Copyright © 2021 Cooper et al. 2021 Cooper et al. https://creativecommons.org/licenses/by/4.0/ This content is distributed under the terms of the Creative Commons Attribution 4.0 International license.

## DISCUSSION

In this study, we manipulated the microbiome of *Daphnia magna* using low doses of targeted antibiotics to examine the impacts of selective suppression on host fitness. We found that aztreonam and sulfamethoxazole, antibiotics targeting abundant bacterial classes in the *Daphnia magna* microbiome, had the largest impacts on host fitness, primarily in affecting host fecundity. However, our results contrast with those of Sison-Mangus et al. (2015) (18) and Callens et al. (2016) (14) in that survival was not impacted by antibiotics here. This is likely due to our use of different antibiotics meant to manipulate taxa differentially in the microbiome, rather than the broad-spectrum suite of antibiotics used to completely suppress the microbiota in those studies. We found that in all of the antibiotic treatments, relative abundance of bacteria was lower than in the control treatment, though only significantly in the antibiotic trio treatment perhaps due to 16S rRNA copy number variation in both suppressed and nonsuppressed taxa (e.g., nonsuppressed taxa having more copies, leading to no decrease in overall 16S abundance). Interestingly, this suppression coincided with an expansion of diversity, with both sulfamethoxazole and the antibiotic trio having significantly higher alpha diversity. This suggests that antibiotic suppression of abundant taxa allows other taxa to flourish, as also indicated by the rare taxa with significantly increased fold changes in these treatments. Our differential abundance analysis shows that even though aztreonam had the fewest changed ASV abundances, host fecundity was still negatively impacted, and across treatments nontarget classes were impacted in unexpected ways potentially due to microbial interactions or off-target antibiotic effects. We also found that shifts in microbiome composition were dependent on environmental conditions, with *Daphnia magna* exposed to the same antibiotics at low temperatures not experiencing the same shifts in microbiome composition as in the control temperature, or the same effects on host fitness by the antibiotic treatments.

Our characterization of the *Daphnia magna* microbiome under standard conditions (19°C, no antibiotics) yields a composition that is similar to that of healthy adult *Daphnia magna* in other studies, though this genotype has been isolated in laboratory culture for >3 years (15, 34, 36). This suggests that our *Daphnia magna* maintained in culture has retained *Daphnia*-specific microbes present during initial field collection, as all culture medium is autoclaved prior to use and algae are grown axenically. We did find that the microbiota of untreated adult *Daphnia magna* in these cultures exhibited a higher relative abundance of *Sphingobacteriia* than in other studies (approximately 48%, compared to 4% to 20%), but other abundant bacterial classes were similar, including *Alphaproteobacteria*, *Betaproteobacteria*, and *Gammaproteobacteria* (15, 34). In particular, we found that species in the *Betaproteobacteria* genus *Limnohabitans* are highly abundant in the *Daphnia* microbiome, consistent with prior work (15, 36–39). While *Limnohabitans* species have strong impacts on *Daphnia* fitness through potential amino acid provisioning (40, 41), they are surprisingly resilient to sublethal antibiotic exposure, as they are not found to be differentially abundant in any of the antibiotic treatments (Fig. 3). Other genera identified here and in other studies include *Pedobacter*, *Emticicia*, and *Acidovorax* (15, 42), among others.

Host fecundity was impacted by treatment with aztreonam, sulfamethoxazole, and the antibiotic trio, and host growth was reduced when treated with the trio as well. Sulfamethoxazole (targeting *Betaproteobacteria* and *Sphingobacteriia*) also delayed age at first reproduction, supporting our hypothesis that suppression of more abundant bacterial classes would have larger impacts on host fitness. Though it is possible that the antibiotics used here have direct effects on host fitness (39), germfree *Daphnia magna* treated with the same antibiotic trio had the same fitness metrics as the *Daphnia* treated here (R. O. Cooper, unpublished data), suggesting that differences in host fitness are mediated through the microbiome. The reduction in host fecundity without a consistent associated decrease in abundance of any particular microbial taxon across treatments suggests that multiple taxa are involved in the functions that benefit host fecundity. It is also possible that the antibiotic treatments reduce host fitness uniquely; for example, the *Perlucidibaca* ASV (ASV 65) is reduced in aztreonam and in the antibiotic trio but not in sulfamethoxazole, while the *Pedobacter* ASV (ASV 12) is reduced in all treatments except aztreonam. Although many of the same taxa were suppressed in erythromycin as in both aztreonam and sulfamethoxazole, many more unique ASVs became more abundant. It is possible that the detrimental effects of taxa reduced across all treatments were countered by increases in these ASVs, which include an *Ensifer* (ASV 18), a *Comamonadaceae* (ASV 9), and a *Sphingopyxis* (ASV 7). These ASVs also appear in the antibiotic trio treatment, but a *Caulobacter* ASV appears as significantly reduced (ASV 66), suggesting that treatment with all three antibiotics simultaneously may counteract the neutral effects observed in the erythromycin-only treatment. Though specific functions encoded by these taxa are unobservable through 16S rRNA sequencing, previous shotgun sequencing work has highlighted some functions in *Daphnia*-associated species. Specifically, metagenome sequencing indicates that a *Pedobacter* species uniquely encodes chitin degradation and sialic acid cleavage and that other species (primarily *Limnohabitans*) may be able to utilize those cleaved sialic acids for amino acid biosynthesis (40). This delay in reproductive maturity and associated reduction in *Pedobacter* in the sulfamethoxazole treatment (and potential counteraction by the increase in *Comamonadaceae* in erythromycin) may indicate that microbe-microbe interactions are affected by targeted antibiotic treatment. Simultaneously, a substantial increase in a *Chitinophagaceae* ASV (ASV 24) is seen in all treatments except aztreonam; *Chitinophagaceae* (like *Pedobacter*) are known chitin degraders and may have detrimental effects on host fitness by utilizing the key material in their host’s carapace. Again, the other ASV changes in erythromycin may counter the potential negative impacts of this taxon on the host, but causality is not understood at this time.

Interestingly, *Gammaproteobacteria* are found in high relative abundances in the *Daphnia magna* gut and could play a role in nutrient acquisition or pathogen protection (14, 15), yet it does not appear that their suppression was a primary driver of changes in host fitness. We hypothesize that this may be due to functional redundancy of taxa found in the *Daphnia* gut, as the indiscriminate filter feeding by *Daphnia magna* exposes gut microbes to a wide array of nutrients (14, 43). Indeed, the abundances and identities of taxa in the *Daphnia* gut vary substantially across studies (16, 18, 27, 39); this variation may allow different taxa not targeted by erythromycin to retain the necessary functions for nutrient acquisition.

Temperature dramatically shifted the microbiome and the fitness of *Daphnia magna*. These changes have been documented across host genotypes and warmer temperatures (12, 27), but to our knowledge, this is the first work to examine the effects of this cold of a temperature (11°C) on the microbiota of this keystone species. *Daphnia magna* raised in cold temperatures survived more, grew less, and had almost no offspring, a well-studied physiological mechanism for actively surviving winter in aquatic ecosystems (44). Correspondingly, the microbiome shifts during this time. In 11°C, *Betaproteobacteria* became even more abundant, comprising >80% of relative abundance. *Sphingobacteriia*, *Flavobacteriia*, and *Gammaproteobacteria* were reduced to <5% relative abundance each. This may be due to cold-induced changes to host metabolic processes like fat storage and processing, which have been shown to shape microbiota composition (45). *Daphnia magna* reduces stearic acid formation at low temperatures but increases monounsaturated fatty acid formation (46), which could select for taxa able to utilize these types of fatty acids. Alternatively, *Betaproteobacteria* may be so important for host fecundity (17) that they must remain in high abundance to ensure they remain for the postwinter reproductive cycle. Though microbiota composition shifted in cold temperatures, it is unlikely that host fitness is mediated by microbiota change. Even with a greater relative abundance of reproductive fitness-promoting *Betaproteobacteria*, *Daphnia magna* in cold temperatures had significantly reduced reproductive fitness, suggesting that temperature directly impacts fitness.

Antibiotics did not affect microbiome composition in cold temperatures as they did under standard conditions. Treatment with aztreonam at 11°C resulted in a microbiome composition nearly identical to that of *Daphnia magna* not treated with antibiotics in 11°C. Though erythromycin does not target *Betaproteobacteria*, this class was reduced in the cold-temperature–erythromycin treatment, suggesting that taxa in the *Daphnia* microbiota may be differentially susceptible to antibiotics depending on environmental factors and based on host physiological responses to the environment. Horizontal gene transfer could play a role in this differential response, as species in the *Daphnia magna* microbiome do encode antibiotic resistance and efflux (40) and this experiment was conducted over a time period long enough to allow antibiotic resistance to establish within species (47). Furthermore, cold temperatures have been shown to increase the abundance of antibiotic resistance genes (48).

Our results suggest that more-abundant classes in the microbiome (in this case, *Sphingobacteriia* and *Betaproteobacteria*) have larger impacts on host fitness than rarer taxa, though previously known important taxa like *Limnohabitans* are seemingly unperturbed by antibiotics. Within-host microbial communities generally have a skewed abundance pattern, where a few species constitute the majority of total abundance but many species are found in low abundances. Some work indicates that abundant taxa contribute to host fitness (49), while other studies indicate that rare, keystone taxa have disproportionate impacts on host fitness (50). However, a general relationship between abundance in the microbiota and benefit to host fitness is hard to untangle in complex systems, and much research in model systems focuses on hosts with single microbial taxa that have significant impacts on host fitness (51). Utilizing animal models like *Daphnia magna* with more than one taxon contributing to host fitness but a relatively simple overall microbial community allows for a greater understanding of the interplay between the microbiota and host. In *Daphnia magna*, more-abundant taxa (e.g., *Limnohabitans*) confer greater benefits to host fitness, primarily through functions that contribute to increases in host fecundity and growth, whereas the loss of rare species had little effect on fitness. At the same time, abiotic conditions can have a much larger effect on host fitness than the microbiome, as demonstrated by the changes in *Daphnia* fitness with temperature not directly mirrored by changes in the microbiota. Our results show that multiple members of the *Daphnia magna* microbiota impact host fitness in different ways and that these impacts must be understood in the broader context of external factors known to directly affect host fitness. Experiments to disentangle the fecundity-reducing effects of different microbes and the underlying mechanisms in this system are necessary. Continued efforts to isolate members of the *Daphnia magna* microbiota paired with single-species and community reinfection experiments, metatranscriptome sequencing to identify important microbial transcripts, and metabolic analysis of the microbial cohorts are all potential methods to identify causal factors in these host-microbe and microbe-microbe relationships.

## MATERIALS AND METHODS

### Daphnia.

This experiment was conducted using *Daphnia magna* clone 8A, taken from Kaimes Farm, Leitholm, Scottish Borders, United Kingdom (52). Stock cultures of *D. magna* clone 8A were maintained in 19°C controlled chambers with a 16-h-light, 8-h-dark light cycle in 400-ml jars with phosphorus- and nitrogen-depleted COMBO medium (53) for multiple generations. Cultures were fed a standardized 0.25 mg C/ml/day using green alga Chlamydomonas reinhardtii (CPCC 243). C. reinhardtii was cultured in COMBO medium. The volume necessary to provide *D. magna* with adequate carbon was calculated using the BioTek Epoch microplate spectrophotometer.

### Experimental design.

Prior to the experiment, 72 *D. magna* animals were moved to 35-ml glass vials with COMBO medium and allowed to mature under controlled conditions. Neonates from the third brood of each adult were pooled within 24 h of birth and randomly assigned to experimental treatments (*n* = 48 per experimental treatment). The experimental treatments consisted of five antibiotic treatments crossed with two temperature treatments, 19°C and 11°C. Antibiotic treatments were as follows: a control treatment with no antibiotics, 500 μg/liter aztreonam, 400 μg/liter erythromycin, 250 μg/liter sulfamethoxazole, and an antibiotic trio consisting of all three antibiotics together at the concentrations listed above. These antibiotic concentrations were chosen on the basis of a pilot experiment showing no short-term toxicity effects on *D. magna* survival but a significant reduction in bacterial abundance (revealed using qPCR with universal bacterial 16S primers). Body size of each *D. magna* was measured from eyespot to beginning of apical spine before placement into the experimental vials. Experimental *D. magna* animals were raised in 35-ml glass vials with COMBO for 21 days. Each vial was checked for survival and fed daily with 0.25 mg C/ml/day of the diet treatment. COMBO with the appropriate antibiotic treatment and dose was replenished every 2 days. Vials were also checked daily for offspring, which were counted and removed if present. At the conclusion of day 21 or upon death, *D. magna* animals were collected from the treatments. Body size was again measured from eyespot to beginning of apical spine to determine growth. *D. magna* animals from each treatment were pooled in sets of 10 in 1.5-ml microcentrifuge tubes for DNA extraction and processing (*n* = 4 per treatment).

### DNA extraction, library preparation, and sequencing.

DNA was extracted from all pooled samples using the Qiagen DNeasy Blood and Tissue Kit using the manufacturer’s spin-column protocol of total DNA from animal tissues (Qiagen, Hilden, Germany). Whole *D. magna* animals were digested with proteinase K for 24 h to ensure that cells within the carapace were lysed but the carapace was not (54). Following extraction, PCR amplification of the V4 region of the 16S rRNA gene was performed using the 515f (5′-GTGCCAGCMGCCGCGGTAA-3′) and 806r (5′-GGACTACHVGGGTWTCTAAT-3′) universal 16S primer pair (55). Amplification consisted of denaturation at 95°C for 3 min, followed by 35 cycles of 95°C for 45 s, 58°C for 30 s, and 72°C for 45 s, and finished with an extension step of 72°C for 5 min. Simultaneously, a subset of samples from each of the antibiotic treatments was prepared for qPCR using the FastStart SYBR green master mix to verify that antibiotic treatments were reducing overall bacterial abundance. Each sample was run in triplicate to ensure amplification was achieved in each sample. All samples were checked for successful amplification using a 1% agarose electrophoresis gel. Samples were then normalized with the SequalPrep normalization plate kit. Prior to sample pooling, sample quality was checked using the Agilent high-sensitivity DNA kit on the Agilent TapeStation and via qPCR with the KAPA library quantification kit. Samples were then pooled and spiked with PhiX DNA. The pooled libraries were then sequenced using the Illumina MiSeq reagent kit v2 (300 cycles) on an Illumina MiSeq. Sequencing was carried out at the Nebraska Food for Health Center (Lincoln, NE, USA).

### Sequencing data processing.

Following sequencing, reads were demultiplexed using Illumina’s built-in MiSeq Reporter software. All reads were then analyzed using DADA2 (56) in R. In DADA2, our pipeline consisted of low-quality (<Q30) read trimming, estimation of read error, dereplication of reads within samples, and chimera removal. Remaining reads were considered amplicon sequence variants (ASVs) and then were assigned taxonomy to the genus level using the RefSeq-RDP database (57). All visualization of ASVs was performed with Phyloseq (58) in R, where reads without a taxonomic assignment at the phylum level and those assigned to “Chloroplast” were removed for visual clarity. All scripts for read processing and visualization are available on GitHub.

### Statistical analysis.

All statistical tests were performed in R. Host *D. magna* life history traits measured as indicators of fitness outcomes included growth, survival, and reproduction. Growth was quantified as the difference between size measurements at the beginning and end of the experiment. Differences in growth among treatments, including the interactions between antibiotics and temperature, were analyzed using an ANOVA. We also used ANOVAs to test for effects of antibiotics and temperature on reproduction, which was measured as number of juveniles per brood and day of first reproductive event (production of the first brood for each individual). Tukey’s HSD *post hoc* tests were conducted to determine which treatments significantly differed from the control treatment. Survival rates among treatments were analyzed using the Cox proportional hazards model. Individuals alive at experiment conclusion were coded as censored. We used the threshold cycle (ΔΔ*C_T_*) method to calculate log fold change in abundance of the 16S rRNA gene among antibiotic treatments, normalizing against the *Daphnia magna* actin gene (forward primer, 5′-CCACACTGTCCCCATTTATGAA-3′; reverse primer, 5′-CGCGACCAGCCAAATCC-3′) and against the control treatment. A PERMANOVA was conducted among treatments on the calculated unweighted UniFrac distances to test the effects of antibiotics and temperature on microbiota composition, and then pairwise comparisons of antibiotic treatments in each temperature were conducted to find treatments with significantly different overall community composition. DESeq2 was used to find differentially abundant taxa among treatments.

### Data availability.

Raw read data are available in the Sequence Read Archive in BioProject PRJNA543842 (https://www.ncbi.nlm.nih.gov/bioproject/PRJNA543842) under accession numbers SRX5866173 to SRX5866265. All code, life history data, and a .rds containing the DADA2-processed read data used in this study are available at https://github.com/reillyowencooper/ab-targeting-daphnia.

## Supplementary Material

Reviewer comments
