# Supplementary figures and images for: Targeted Manipulation of Abundant and Rare Taxa in the Daphnia magna Microbiota with Antibiotics Impacts Host Fitness Differentially
Source: mSystems. 2021 Apr 6;6(2):e00916-20. doi: 10.1128/mSystems.00916-20 (PMC8546987; doi:10.1128/mSystems.00916-20)

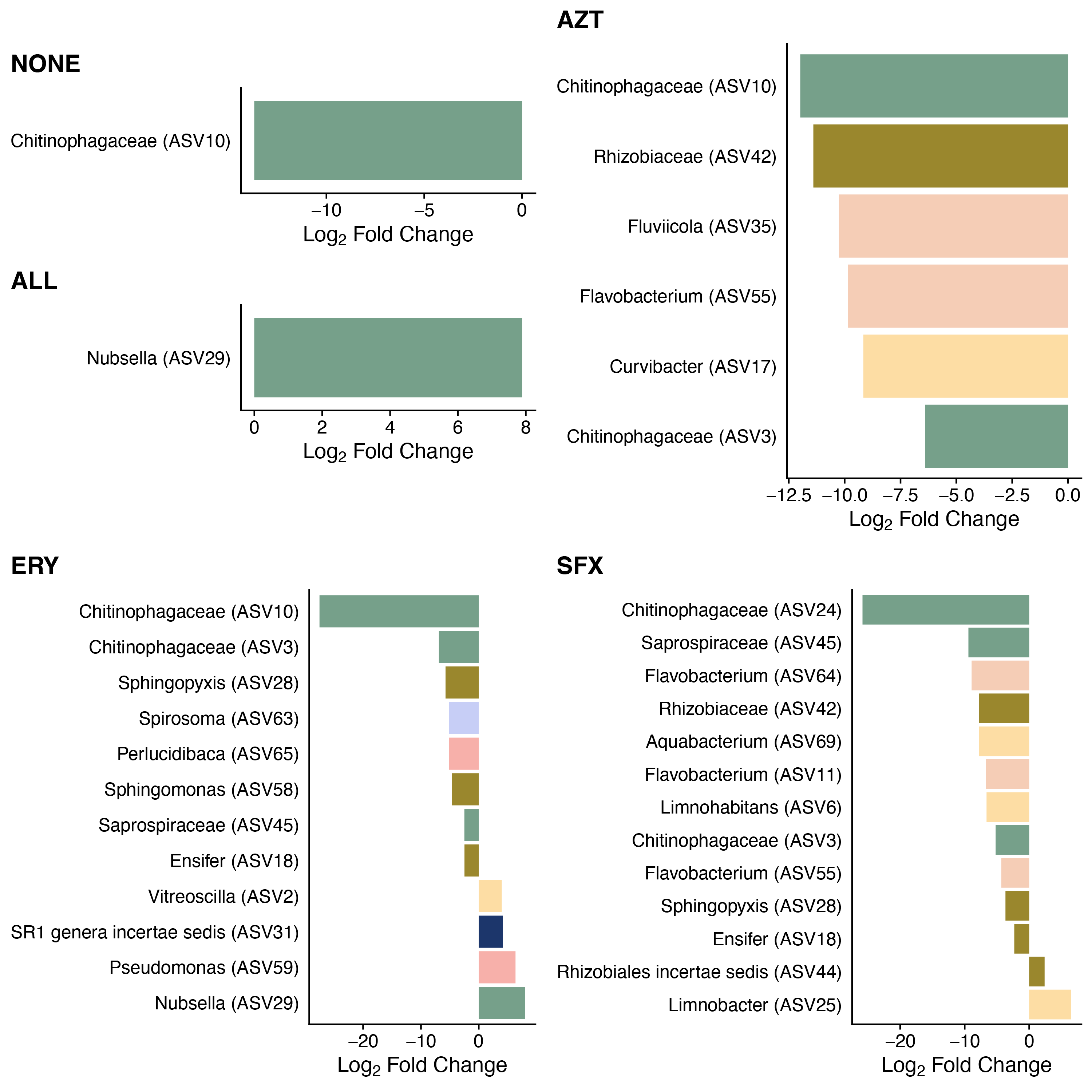

Supplement: FIG S1 [file msystems.00916-20-sf001.tif]

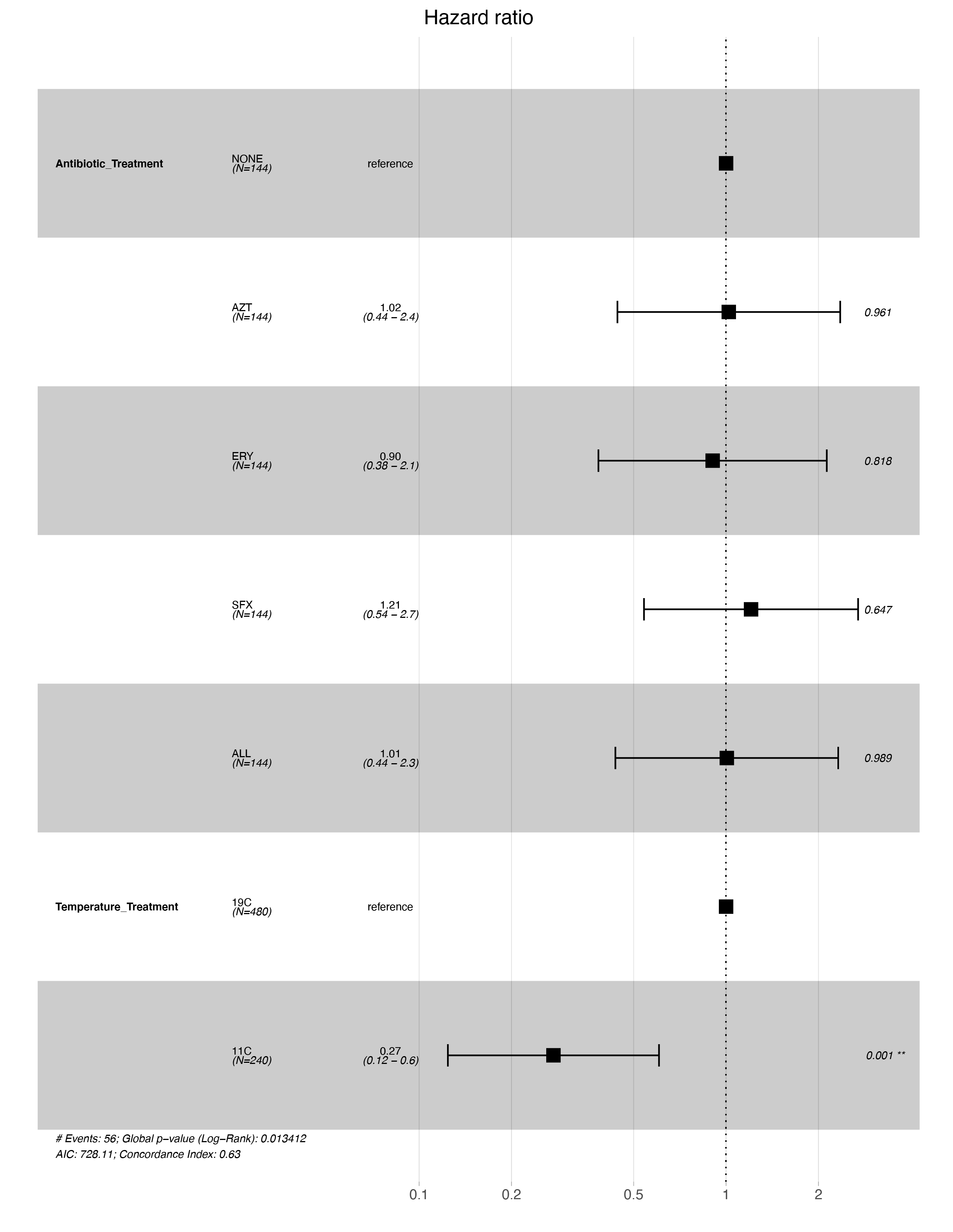

Supplement: FIG S2 [file msystems.00916-20-sf002.tif]
